# Key genes and co-expression modules involved in asthma pathogenesis

**DOI:** 10.7717/peerj.8456

**Published:** 2020-02-03

**Authors:** Yuyi Huang, Hui Liu, Li Zuo, Ailin Tao

**Affiliations:** 1 The State Key Laboratory of Respiratory Disease, Guangdong Provincial Key Laboratory of Allergy & Clinical Immunology, The Second Affiliated Hospital of Guangzhou Medical University, Guangzhou, China; 2 School of Basic Medical Sciences, The Sixth Affiliated Hospital of Guangzhou Medical University, Guangzhou, China; 3 The Interdisciplinary Biophysics Graduate Program, The Ohio State University, Columbus, OH, USA; 4 College of Arts and Sciences, University of Maine Presque Isle Campus, Presque Isle, ME, USA

**Keywords:** Asthma, WGCNA, Machine learning, Pathology, Endocyte

## Abstract

Machine learning and weighted gene co-expression network analysis (WGCNA) have been widely used due to its well-known accuracy in the biological field. However, due to the nature of a gene’s multiple functions, it is challenging to locate the exact genes involved in complex diseases such as asthma. In this study, we combined machine learning and WGCNA in order to analyze the gene expression data of asthma for better understanding of associated pathogenesis. Specifically, the role of machine learning is assigned to screen out the key genes in the asthma development, while the role of WGCNA is to set up gene co-expression network. Our results indicated that hormone secretion regulation, airway remodeling, and negative immune regulation, were all regulated by critical gene modules associated with pathogenesis of asthma progression. Overall, the method employed in this study helped identify key genes in asthma and their roles in the asthma pathogenesis.

## Introduction

Asthma is a complex disease with diverse underlying pathological mechanisms with both the young and the elderly (Hasegawa et al., 2017; Li et al., 2016; Lotvall et al., 2011). Bronchial hyperresponsiveness, airway remodeling (Movassagh et al., 2016), abnormal hormone secretion (Newton et al., 2017), and chronic airway inflammation (Parulekar, Diamant & Hanania, 2017) are some of the major clinical features of asthma.

For most patients, bronchodilator or inhaled corticosteroids have been effective in treating asthmatic symptoms. However, some patients did not respond to these therapies (Swedin et al., 2017). Patients with high Th2 cytokines were not responsive to inhaled corticosteroid (Parulekar, Diamant & Hanania, 2017). Interestingly, some individuals had favorable responses to anti-IL-13 and anti-IL-5 treatments (Brightling et al., 2015; Corren et al., 2011; Hanania et al., 2015). These studies suggested that it is worthwhile to research the key genes and the pathogenic mechanisms of asthma. As such, one tool that can help researchers with the analysis of the relationships between key genes and the pathogenic mechanism is weight gene co-expression network analysis (WGCNA) (Li et al., 2016). The WGCNA method has been widely used in recent years (Abu-Jamous & Kelly, 2018; Liu et al., 2017; Mack et al., 2018; Radulescu et al., 2018). Instead of linking thousands of genes to the disease, this technology focuses on relationship between gene modules and disease traits. Through WGCNA, hidden biological models of the disease can be discovered (Giulietti et al., 2018; Vella et al., 2017).

Machine learning has shown great promise for mining linear or non-linear relationships in high-dimensional data through supervised (Ahuja et al., 2019), unsupervised (Tshitoyan et al., 2019) or semi-supervised methods (Tarbell & Liu, 2019). It can also reflect the properties of high dimensional data. Because of such property, it can effectively reduce data dimension and improve data understanding. Thus, it can be useful for the analysis of transcriptomic data with high dimension, large numbers of genes and complex relationships (Bogard et al., 2019; Kachroo et al., 2019). Machine learning algorithms can also be useful at classification tasks. Hirai et al. (2017) employed machine learning to group patients with both asthma and chronic obstructive pulmonary disease (COPD) according to their clinical features . The results revealed three clusters that belonged to the asthmatic patients and one cluster that belonged to the COPD patients. Thus, it seems that the machine learning algorithm can effectively distinguish asthma from COPD. Furthermore, there are different phenotypes and properties associated with asthma. The selected feature gene set lacks biological significance with unclear pathways of feature genes; thus, analysis of other biological networks is needed to confirm.

This study aims to improve the assessment of pathogenic mechanisms by incorporating merits of machine learning and WGCNA. In addition, the study is designed to discern key genes in asthma and to understand their role in the asthma pathogenesis.

## Materials and Methods

### Weighted gene co-expression networks analysis

WGCNA was used to identify gene co-expression networks associated with clinicopathological factors of asthma. For example, the GSE43696 dataset contains all clinical information of asthma severity in the Gene Expression Omnibus database. In total, 108 samples identified the severity of asthma and 30,723 genes were included. As module identification required intensive computation, the top 5,000 genes with highest expression variance and closely connected were selected to construct the weighted gene co-expression network. Then, a correlation matrix was constructed using calculated pairwise Pearson Correlations among all genes. To achieve a scale-free network, *β* = 8 was used as the proper soft-thresholding power to convert the pairwise correlation into an adjacency matrix of connection strengths (connection strength = —correlation—^*β*^). To identify gene modules, a dissimilarity matrix with via a dynamic tree-cutting algorithm was used based on the topological overlap measure. All gene modules were allocated with appropriate colors. The gene modules with similar expression profiles were also merged.

### Annotation and enrichment analysis of gene modules

To explore the biological functions of gene modules, Gene Ontology (GO) term enrichment analyses were performed to describe module function and identify relationships between these gene modules using the *Gostats* package in R (Falcon & Gentleman, 2007). The hypergeometric test was used to estimate the GO term association, while the *P* value was adjusted by the Benjamini–Hochberg method. Gene modules were named according to the most significant GO enrichment.

### Calculation of module-trait correlations

An advantage of co-expression network analysis is the capacity to integrate external information. The correlations between gene modules and asthma severity were determined in this study. The significance of the module could be determined as the average absolute gene significance index. After the aforementioned procedures, the color intensity was identified to be proportional to the disease status.

### Development of a random forest model and feature selection

A tenfold cross validation (CV) technique was used to build and verify the 108 samples. The entire dataset was randomly divided into 10 subsets, with approximately 10% test data. In each round of CV, 9 subsets were used to train the model and to predict the outcome of tested subset. This process was performed 10 times until each subset was fully tested. The statistical indicators, such as out of the bag (OOB) estimates of error rate between the CV predictions and the observed values, were used to evaluate the prediction accuracy of the model. Then, recursive feature elimination based on random forest analysis was used to select the feature genes associated with asthma severity (Nguyen & Ohn, 2006). Recursive feature elimination random forest algorithm is a built-in feature selector, which follows the backward elimination method. The embedded learning algorithm is the random forest, which identifies the most related genes for a disease by feature selection. In this study, all undecided features were assumed to be irrelevant. The algorithm reinitialized feature genes after every iteration.

### Statistical analysis

Statistical significance was determined using the *t*-test and One Way ANOVA test with R software. *P* < 0.05 was considered as a statistically significant difference.

## Results

### Construction of weight gene co-expression network

**Figure 1:**
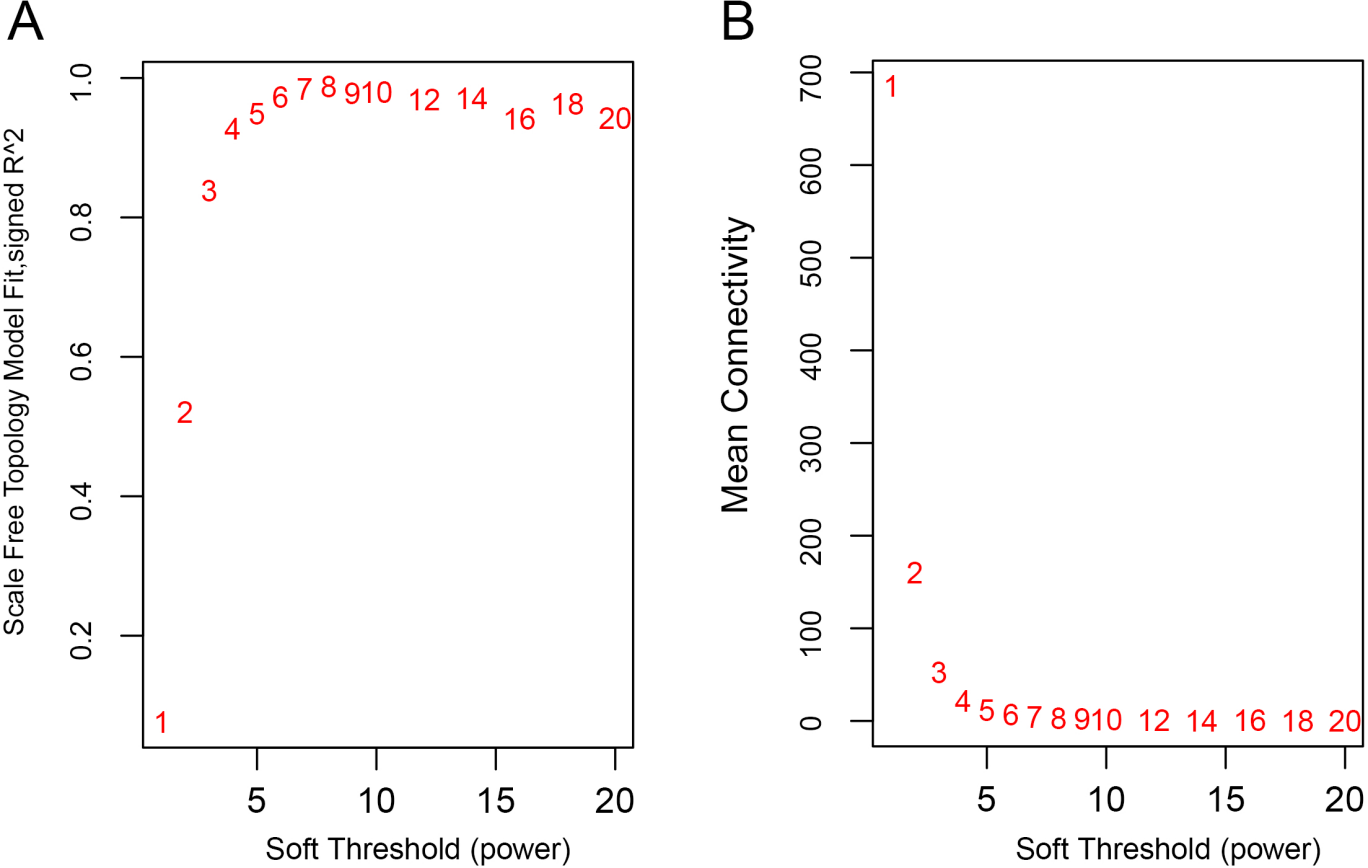
Determination of soft-thresholding power. (A) Analysis of the scale-free fit index for various soft-thresholding powers (*β*). (B) Analysis of the mean connectivity for various soft-thresholding powers.

**Figure 2:**
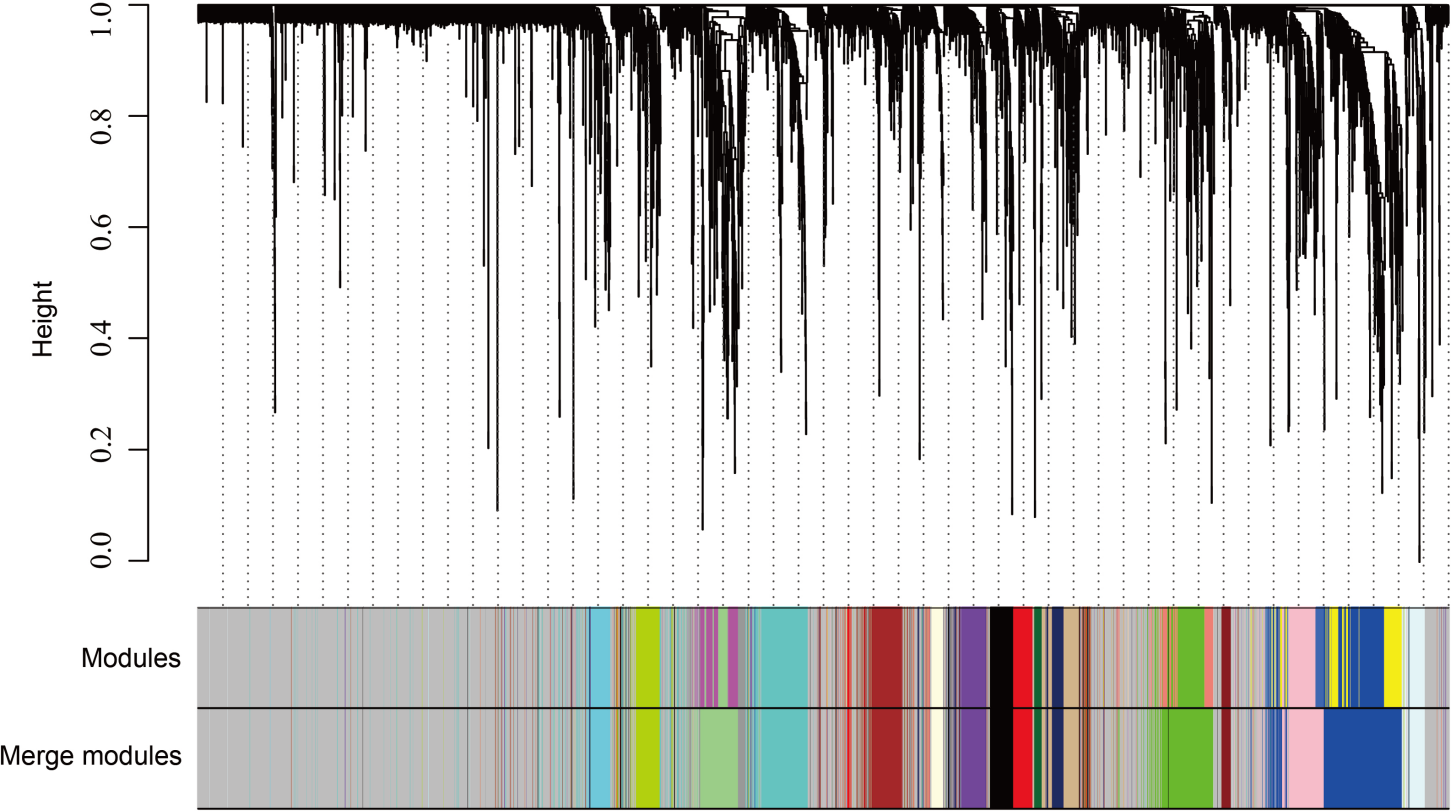
WGCNA correlation network results in asthma. Clustering dendrogram of species, with dissimilarity determined by topological overlaps, along with assigned module colors. Weighted gene co-expression network analysis (WGCNA) can be used to group genes into 18 different gene modules based on their co-expression patterns.

**Figure 3:**
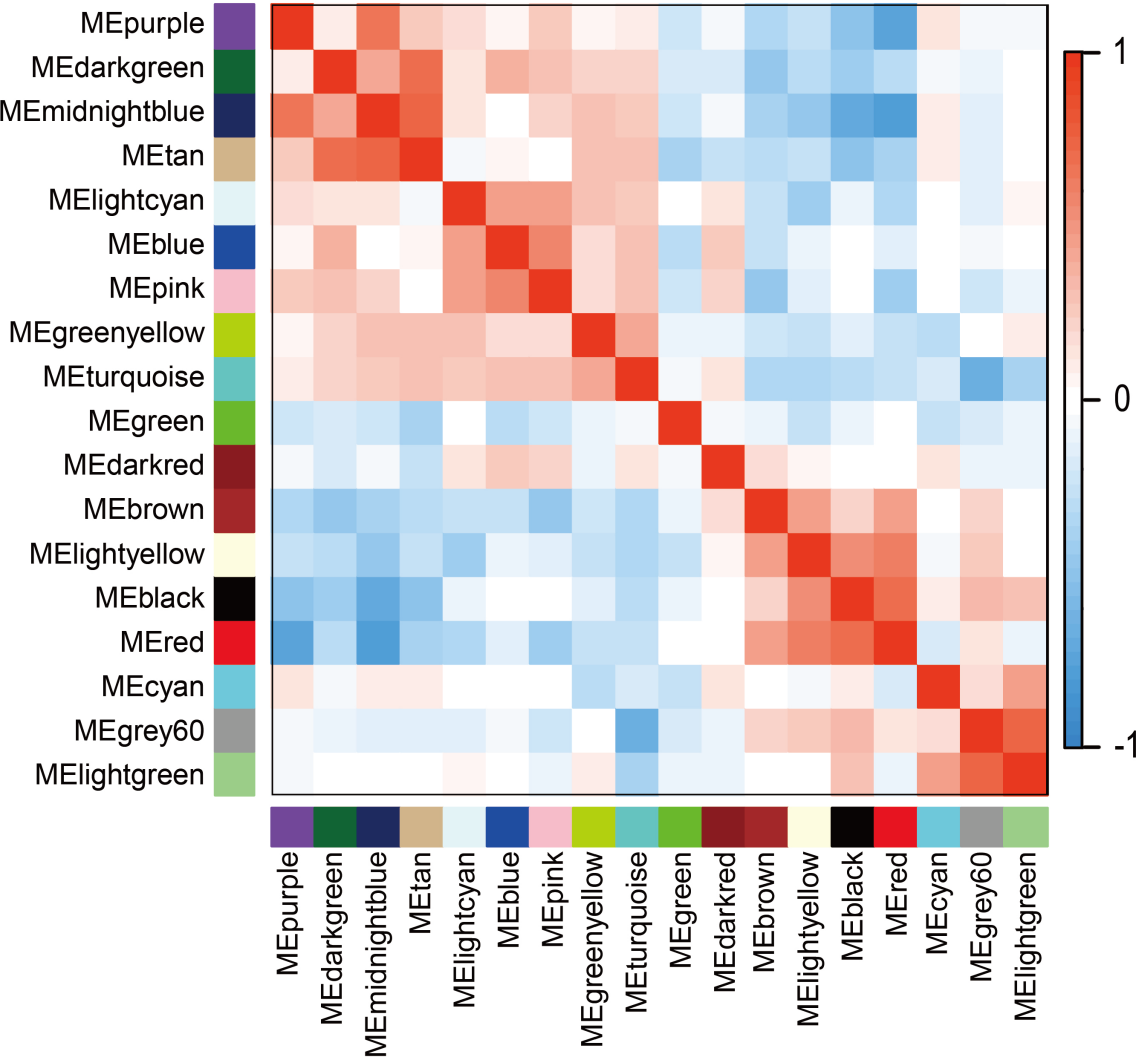
Module eigengene adjacency heatmap. Module-eigengenes (ME) are defined as the first principal component of a coexpression module matrix. The heatmap shows the relatedness of 18 co-expression gene modules identified by WGCNA (red, positive correlation; blue, negative correlation). Color scale indicates the range of correlation coefficients. The correlation coefficient is between −1 and +1, where ±1 indicates the strongest possible correlation and 0 indicates the weakest possible correlation.

The WGCNA was performed to identify the gene co-expression networks associated with the clinicopathological factors for asthma. The asthma dataset, namely GSE43696, was adopted from the GEO database (Voraphani et al., 2014). It worth noting that soft threshold is a key parameter for WGCNA to measure gene relationship. Adjusting soft threshold can convert simulated gene network into justified biological network. In this regard, when soft thresholding is adjusted to value 8, the simulated gene network has the optimal correlation to the real biological network (Fig. 1). After this soft threshold of 8 was implemented, 18 significant gene modules were thus detected (Fig. 2). The relationships between gene modules are shown in Fig. 3. The results indicated that some gene modules strongly correlated with each other, such as red and black, midnight blue and tan, tan and dark green, as well as midnight blue and purple.

### Gene ontology and pathway enrichment analysis of gene modules associated with asthma severity

The biological functions of the gene modules associated with asthma severity were explored by GO term enrichment analysis. For these module genes, the significant enriched terms in the GO and pathway databases were the followings: “regulation of hormone secretion”, “actin filament organization”, “negative regulation of immune response”, “regulation of blood coagulation”, “G-protein-coupled receptor signaling pathway”, “epithelial-to-mesenchymal transition”, and “lipid homeostasis” (Table 1). Thus, the enriched terms in the annotation systems were related to different pathological mechanisms.

### Calculation of module-trait correlations in asthma severity

**Figure 4:**
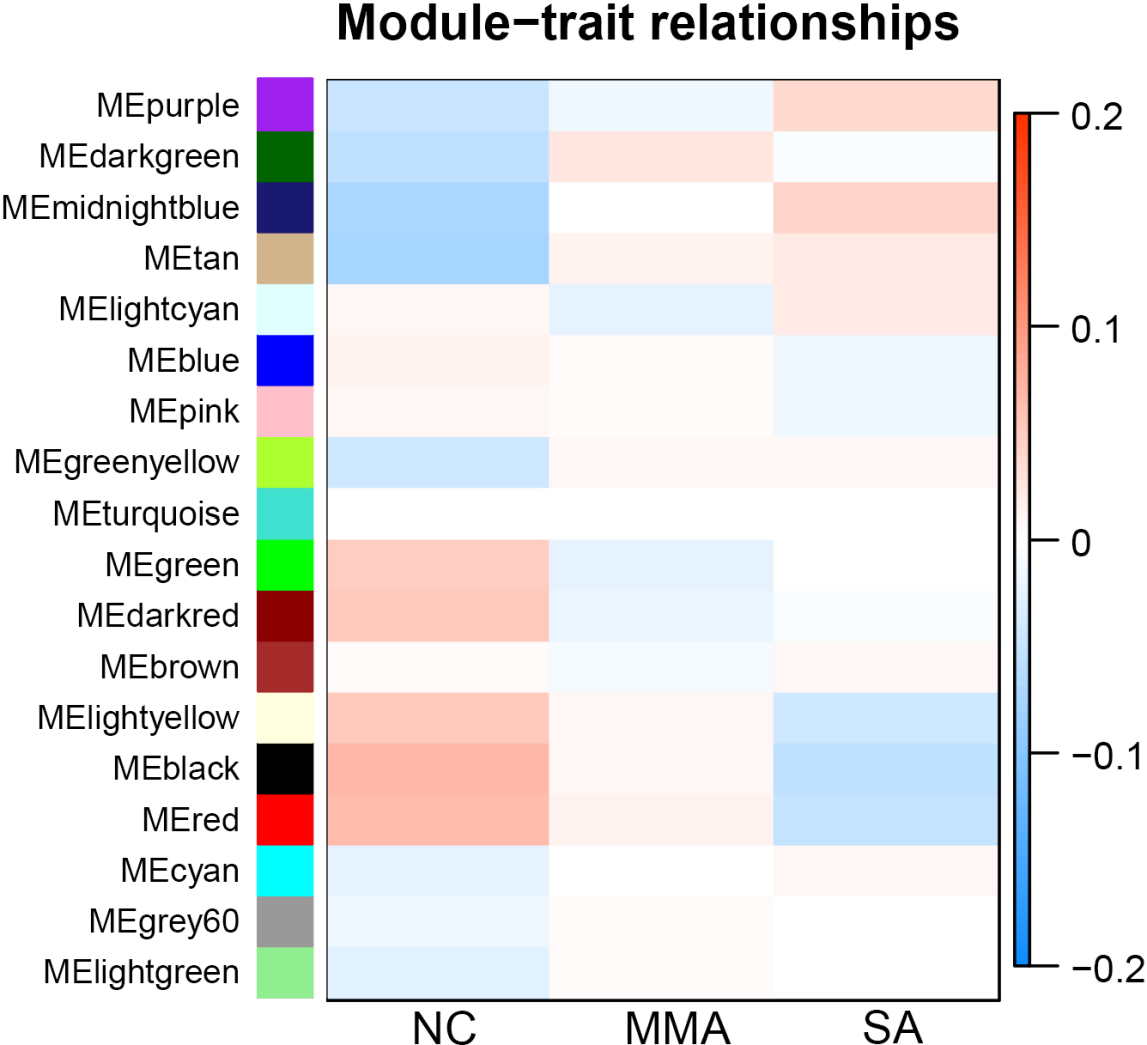
Correlation matrix between each module and severity levels of asthma. Each module is assigned to a color. Each module is tested for correlation with the severity levels of asthma (normal control, mild-moderate asthmatic, and severe asthmatic). Cell colors encode correlation coefficients (red, positive correlation; blue, negative correlation). Color scale indicates the range of correlation coefficients.

For each module, correlations between gene expression and asthma severity were calculated. Multiple gene modules were found to be associated with asthma severity after WGCNA, each named after their representative color: black, red, tan, dark red, dark green, light yellow, and midnight blue (Table 1). The significance of module-trait relationship is shown in Fig. 4. The result showed that different pathological mechanisms had varying degrees of change from mild to severe asthma. Some biological functions decreased, including hormone release, airway remodeling, and activation of the G-protein–coupled receptor. Other biological functions increased, including blood coagulation and angiogenesis, transition from epithelial to mesenchymal, and negative regulation of immune response. These findings suggested that it was difficult to choose the determinant pathological mechanisms associated with asthma severity from a bunch of statistical pathological mechanisms.

**Table 1:** GO enrichment analysis of gene modules. Statistical signicance was determined by the GOstats package with R software.

Color	Pathological mechanism	*P*-value
Black	Actin filament organization	0.002908
Red	Regulation of hormone secretion	0.005152
Lightyellow	G-protein coupled receptor signaling pathway	0.005676
Midnightblue	Regulation of blood coagulation	0.006019
Tan	Negative regulation of immune response	0.008272
Darkgreen	Lipid homeostasis	0.024375
Darkred	Epithelial to mesenchymal transition	0.025982
Purple	Regulation of angiogenesis	0.045193

### Selection of feature gene associated with asthma severity

**Figure 5:**
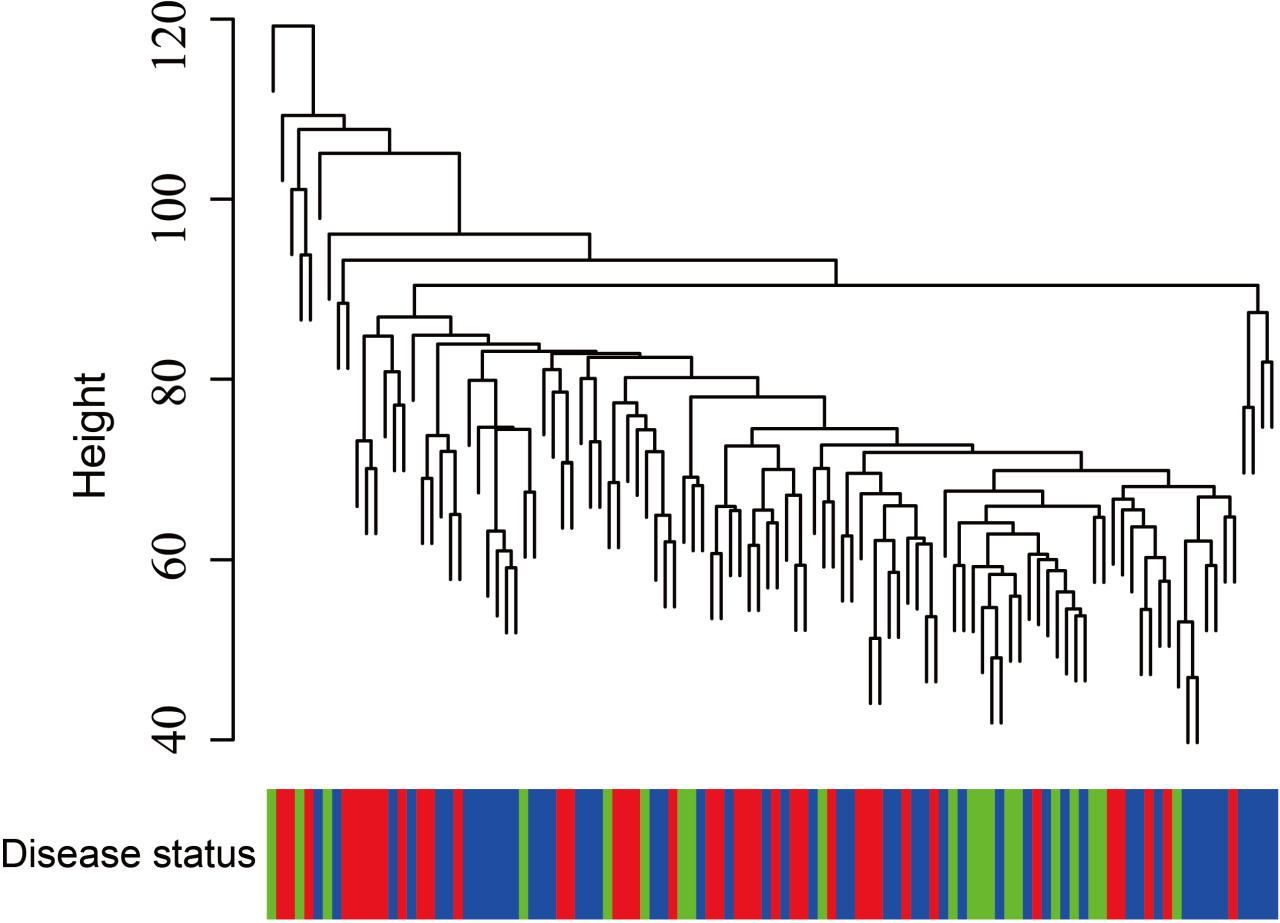
Clustering of asthma samples. The clustering was based on the expression data of GSE43696, which contained 38 SA, 50 MMA and 20 normal samples. The top 5,000 genes with the highest SD values were used for WGCNA analysis. The color intensity was proportional to disease progressive status (normal control, mild-moderate asthmatic, and severe asthmatic).

**Figure 6:**
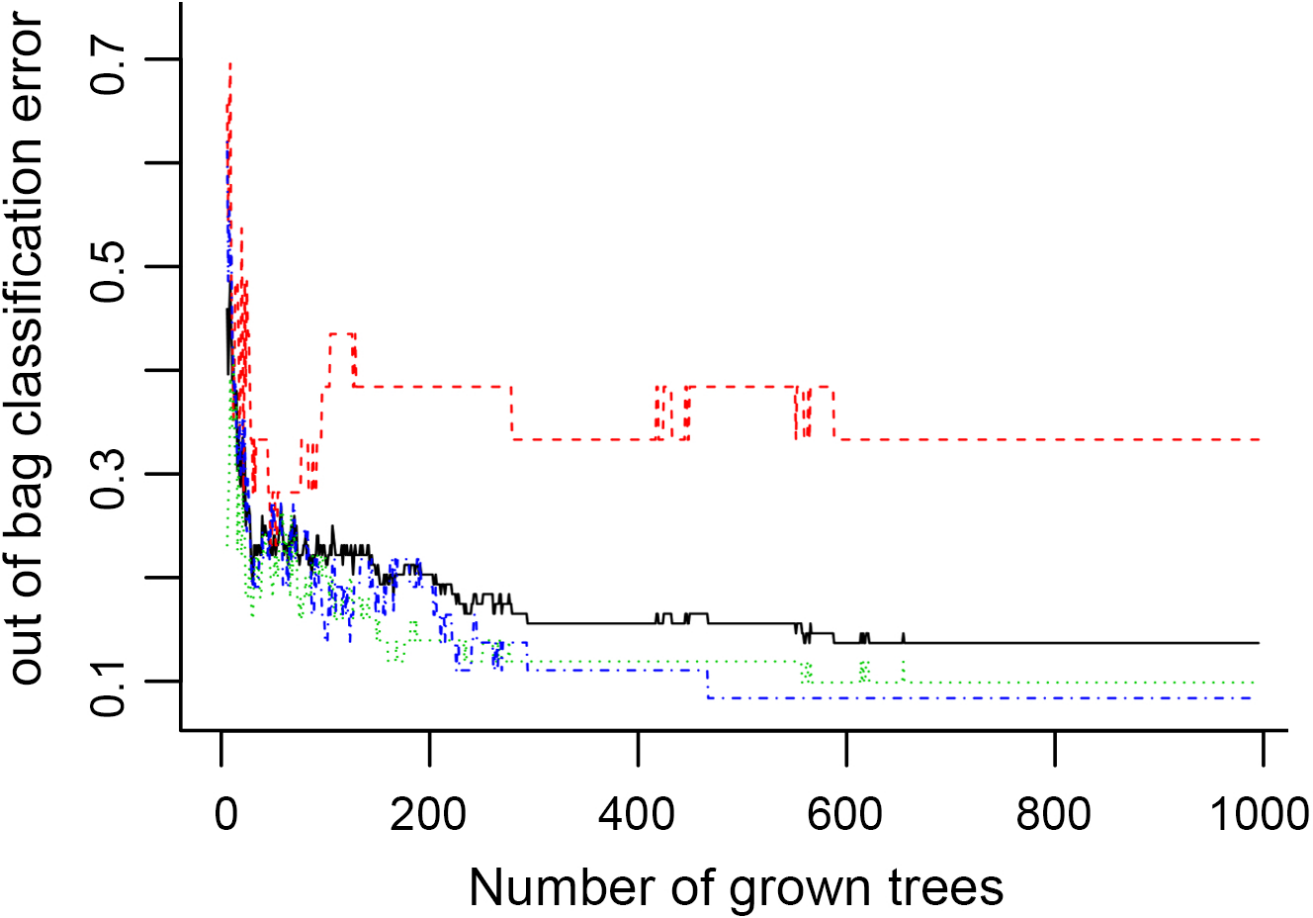
The number of trees grown for random forest (RF) models of asthma dataset before variables ranked by permutation accuracy importance.

**Figure 7:**
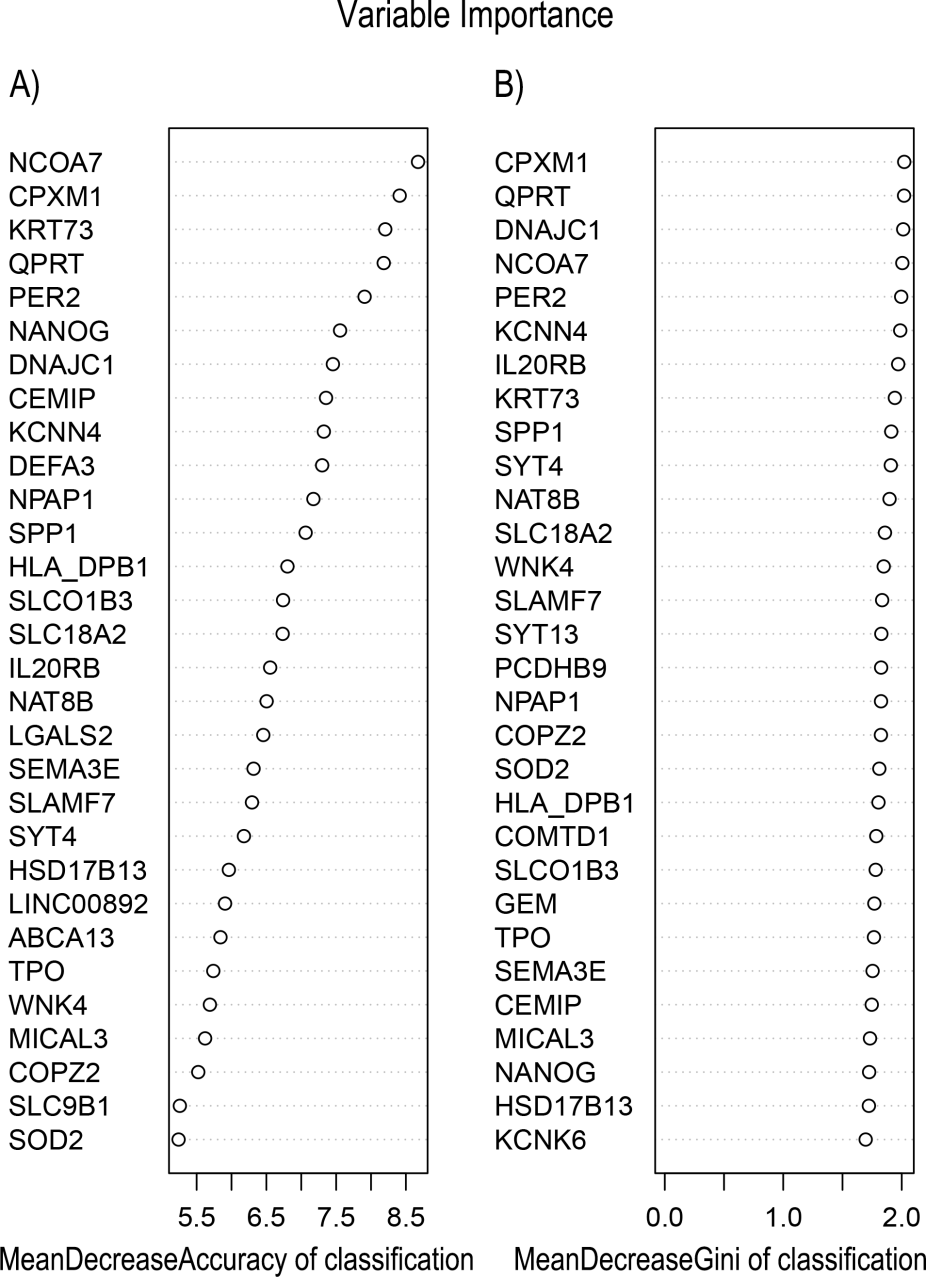
Variable Importance plots obtained from Random Forest in R show the feature gene related to severity levels in asthma ranked on the basis of (A) Mean Decrease in Accuracy and (B) Mean Decrease in Gini coefficients. MeanDecreaseGini: Gini is defined as “inequity”. Gini importance measures the average gain of purity by a given feature gene. If the gene is useful, it tends to split mixed labeled nodes into pure single class nodes. MeanDecreaseAccuracy: a mean decrease in classification accuracy measures the average increase in misclassification in absence of the given feature gene from the gene set.

**Figure 8:**
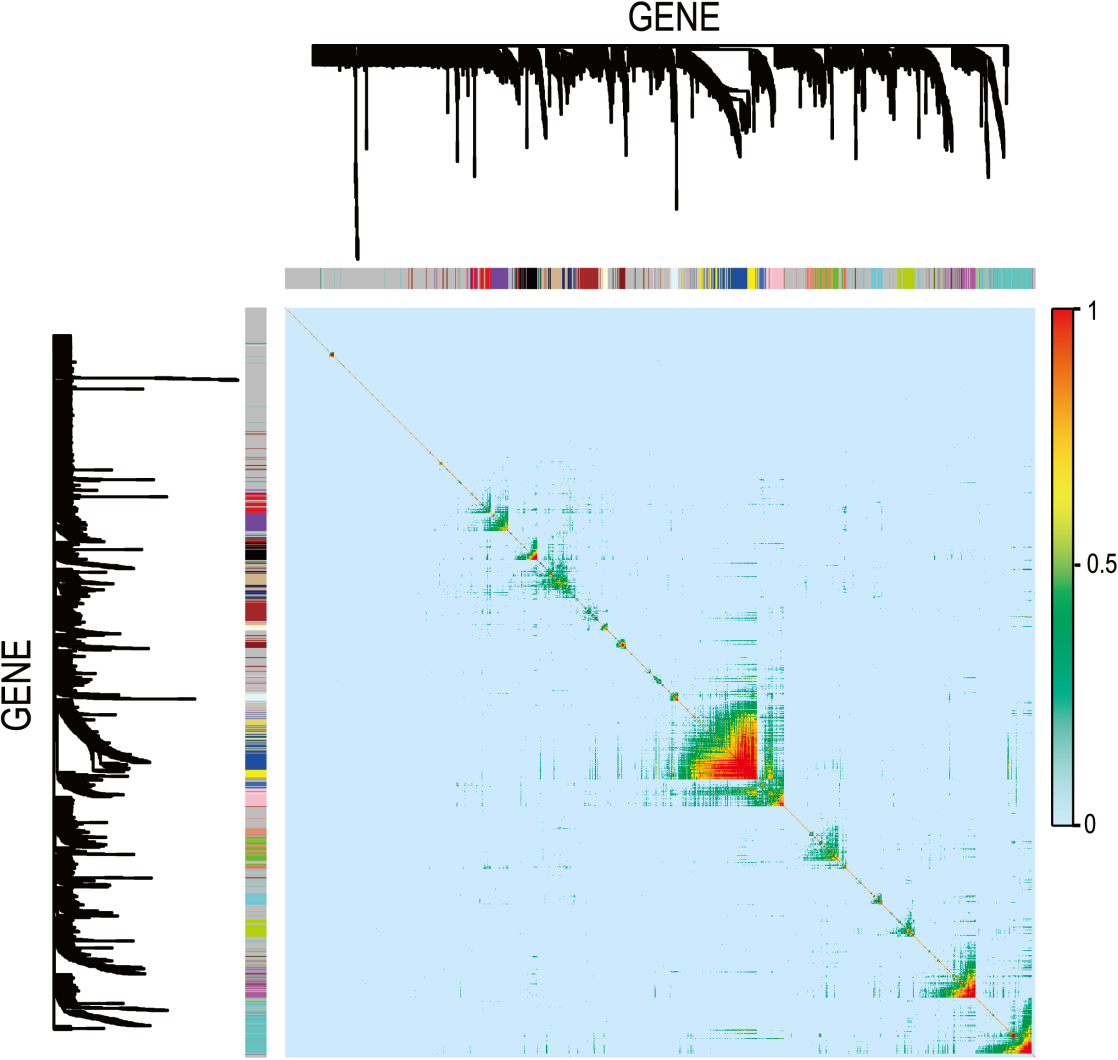
Visualizing expression pattern gene network in asthma using a heatmap plot by WGCNA. The heatmap depicts the TOM among differentially expressed genes in the analysis. Light color represents low overlap and progressively darker red color represents higher overlap. Genes that could not be assigned to a module are labeled gray.

**Figure 9:**
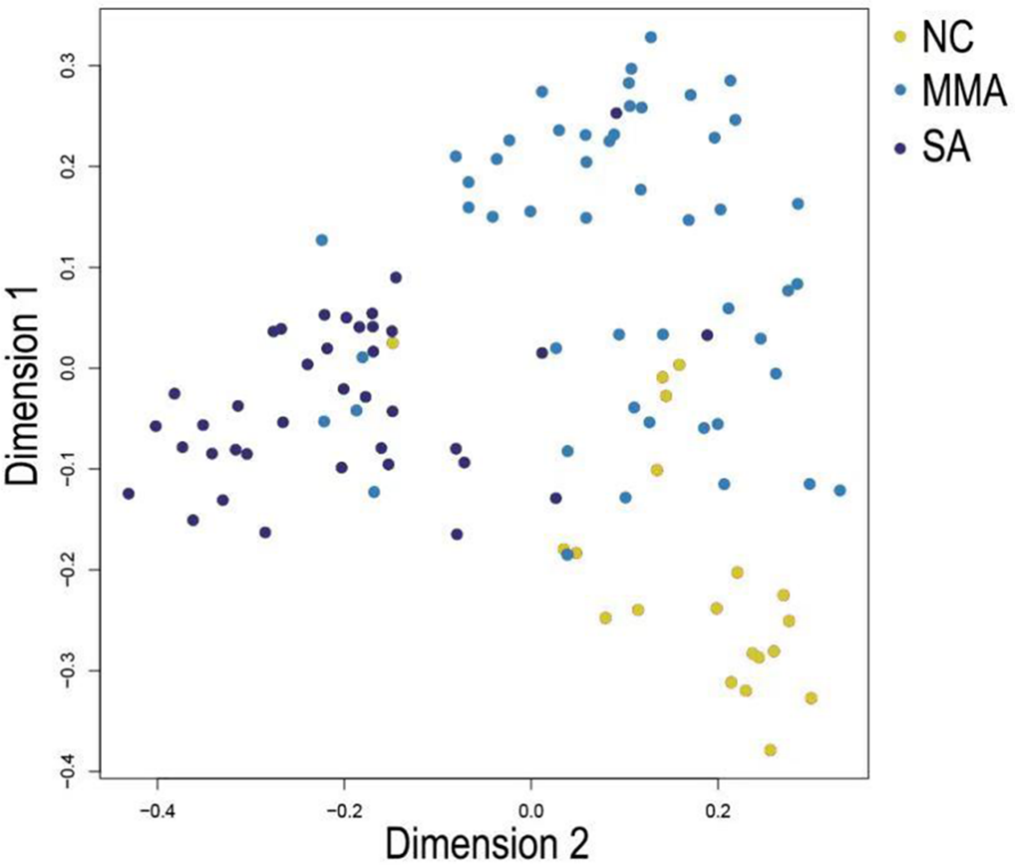
Multi-dimensional scaling (MDS) plot shows differentiation among severity of asthma patients by random forest classifier constructed from 37 feature genes.

In Fig. 5, when all the genes were used to classify the samples, the clustering results were dispersed, and justified division of the asthma severity could not be obtained. While thousands of genes are involved, these could be just random noises. The feature gene selection created by machine learning method can extract effective information from the noise background. Thus, the optimal feature gene set can be readily formed. Accordingly, feature gene selection that was based on random forest analysis (Oussar, 2003), can be used to select the feature gene associated with asthma severity. Figure 6 illustrates that stable results could be obtained when the number of tree models was 1,000 pre-training. 37 stable genes were retained after three replicates random forest analysis, which were ranked as an important factor in the division of asthma severity (Fig. 7). The study found that the strong interaction between SEMA3E and WNK4 and between COMTD1 and DNAJC1 by correlation analysis (Fig. 8). The cross-validation results show that the 37 feature genes are apparently superior to whole genes pool in the parameter OOB estimate of error rate (15.74% vs 51.85%) (Table 2). These 37 feature genes can accurately distinguish different severity in asthma (Fig. 9), showing an essential role in asthma severity.

### Combination of feature genes selection and WGCNA

**Figure 10:**
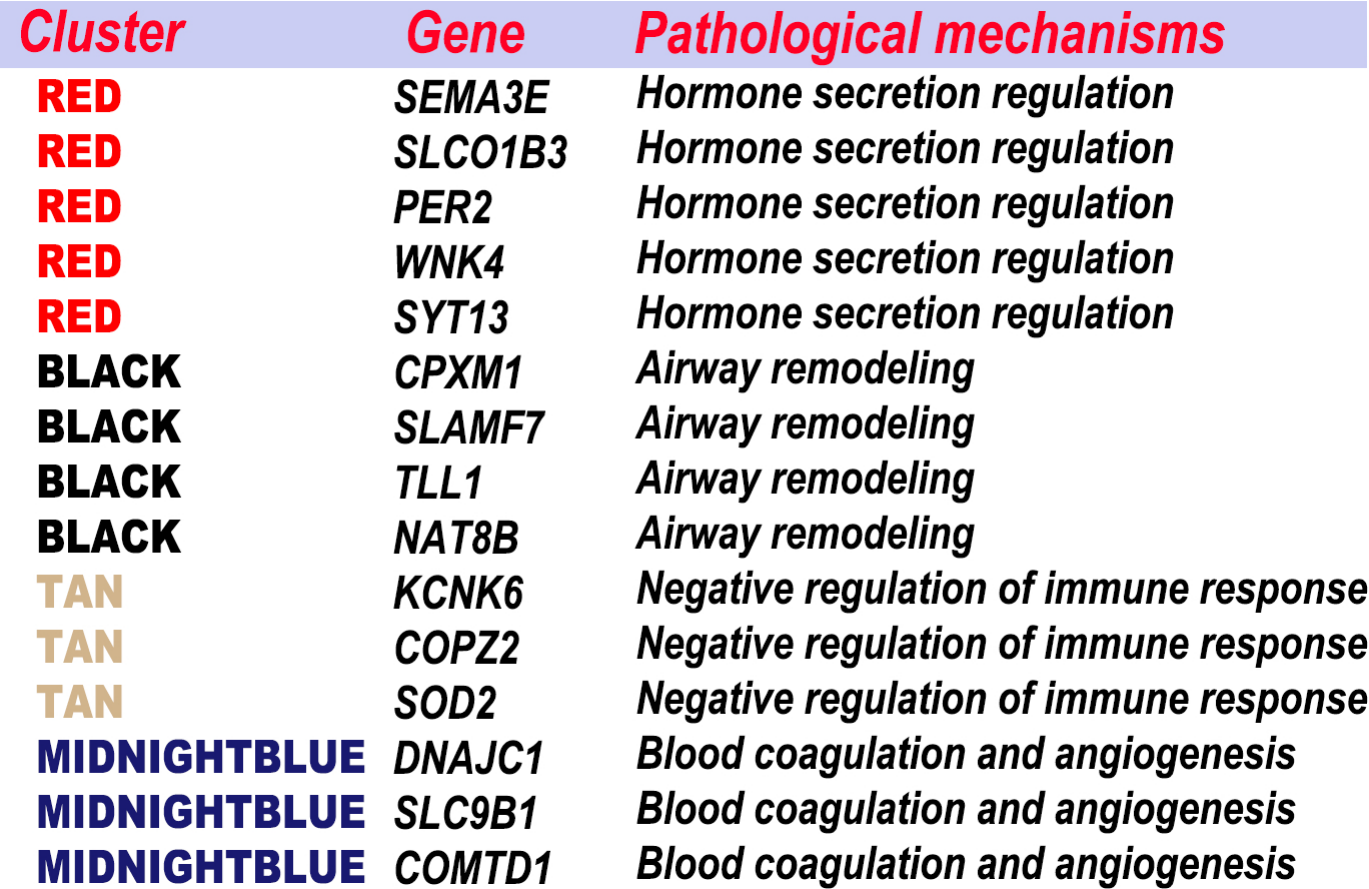
Combination the result of feature selection and WGCNA. The feature genes were clustered according to the specific pathological process.

The feature genes, which were screened out based on the random forest analysis, were endowed with clinical significance using WGCNA. Then, the feature genes were clustered according to the specific pathological process (Fig. 10). Most genes were classified in a few pathogenese. Some feature genes were classified in the hormone secretion regulation, including SEMA3E, PER2, WNK4 and SYT13; some in the airway remodeling, including CPXM1, TLL1 and NAT8B; some in the blood coagulation and angiogenesis, including DNAJC1, COMTD1, and SLC9B1; and some in the negative regulation of immune response, including KCNK6, COPZ2, and SOD2. Overall, 22 of the 37 genes were not classified in the WGCNA gene module or significant gene modules associated with asthma severity. 15 feature genes from the aforementioned four categories accounted for the vast majority. Nine of these were previously implicated in asthma or other respiratory diseases. These results indicated that hormone secretion regulation, airway remodeling, and negative regulation of immune response, can be playing a key role in asthma severity in current settings. The feature genes are also an important factors to be considered in the pathogenesis and classifications.

## Discussion

In this study, a comprehensive analysis of key genes and pathological processes associated with asthma severity is carried out in expression profiling with 108 samples. The goal of this study is to provide insights into the relationship between disease biology and the development of asthma. The findings address the shortage of objectivity in disease pathological diagnosis and in guiding the clinical treatment applications.

Machine learning feature selection has been widely used due to its objective assessment and optimal accuracy in artificial intelligence (Li et al., 2017; Nidheesh, Abdul Nazeer & Ameer, 2017). The feature genes for the development of asthma are screened out using machine learning feature selection. 37 genes associated with asthma development are all retained after feature selection of machine learning. These feature genes can accurately distinguish different severity of asthma (Fig. 9), playing an essential role in asthma. In previous analysis of this asthma dataset (GSE43696), thyroid peroxidase (TPO) plays an important role in asthma (Voraphani et al., 2014). TPO and its metabolome drives nitrative stress in severe asthma. Similarly, TPO is attributed to the feature gene set after the screening of feature genes in our study. These gene sets can effectively distinguish severe asthma patients from the control. However according to the classification, feature gene contribution shows that TPO is low-ranked in the feature gene set. Thus, asthma, a complex disease, is more likely to be the result of multi-gene interactions.

Due to the multiple functions of genes, it is challenging to locate the exact asthma mechanism (Cao et al., 2015; Li et al., 2017; Singh & Sivabalakrishnan, 2015). Hence, WGCNA, based on biological and medical background, is used to endow these genes with clinical significance and cluster the feature genes according to the specific pathological process. However, WGCNA, being considered as a correlation analysis, cannot solve all problems, but needs to combine other appropriate methods. (Li et al., 2016).

This study combines machine learning and WGCNA for the improvement of assessment regarding pathogenic mechanisms. After these processes, the feature genes that played a role in asthma severity can be classified into three major pathological processes: hormone secretion regulation, airway remodeling, and regulation of immune response. These pathological processes and related feature genes can determine the development of asthma. As a result, some genes screened out have been actually reported to be associated with respiratory diseases, such as the gene of superoxide dismutase 2 (SOD2). Previous study identifies production of H_2_O_2_ as a key driver of reactive oxygen species (ROS) that leads to lung damage in asthma. SOD2 could promote the development of inflammation since it is a generator of H_2_O_2_. On the contrary, in our study, superoxide dismutase 2 (SOD2), is identified as an inhibitor of immune responses, as validated by the latest research (Seo et al., 2019). Codonopsis lanceolata extract (CLE) has anti-asthmatic and anti-inflammatory effects. Treatment with CLE enhanced the expression of SOD2, which is related to mitochondrial ROS (mROS) scavenge and Th2 cell regulation. It indicates that CLE has a potential to enhance the immune-suppressive property by regulating mROS scavenging through SOD2. Furthermore, previous studies have reported that SOD2 can be used as an anti-inflammatory agent due to its ROS scavenging capacity (Li & Zhou, 2011). The SOD2 expression level is decreased in multiple diseases, including cancer, neurodegenerative diseases, and psoriasis. The reduction of SOD2 mRNA expression was also observed in our study from mild to severe asthma. Therefore, SOD2 should be identified as an inhibitor of immune response. In addition, the above results also prove the effectiveness of our method.

**Table 2:** The Confusion matrix of estimated error rates with a 10-fold cross validation (CV) for feature genes. (A) Out-of-bag (OOB) estimate of 5000 dierential expression genes is 51.85%. (B) Out-of-bag (OOB) estimate of 50 feature genes is 15.74%. Out-of-bag (OOB) estimate: also called out-of-bag (OOB) error, is a method for calculating prediction error of random forests. It uses bootstrap aggregating (bagging) to classify the sample data.

A. Out-of-bag (OOB) estimate of 5000 genes is 51.85%				
	NC	MMA	SA	Error rate
NC	0	19	1	1
MMA	0	44	6	0.12
SA	0	30	8	0.79

**B. Out-of-bag (OOB) estimate of 37 feature genes is 15.74%**				
	NC	MMA	SA	Error rate
NC	13	6	1	0.35
MMA	1	44	5	0.12
SA	0	4	34	0.11

In summary, our result identify that hormone secretion regulation, airway remodeling, and negative regulation of immune response are all the key factors in the development of asthma severity. Meanwhile, feature genes and their corresponding pathological mechanisms associated with asthma severity are well defined. Overall, the method presented in this study would help narrow down areas where scientists need to concentrate and understand better how key genes are involved in pathophysiological processes of asthma severity. It can also be useful to serve as a basis for classifying asthma phenotypes.

##  Supplemental Information

10.7717/peerj.8456/supp-1 Supplemental Information 1 R source code for gene module and WGCNA

10.7717/peerj.8456/supp-2 Supplemental Information 2 R source code for feature gene screening

10.7717/peerj.8456/supp-3 Supplemental Information 3 Gene module information of asthma development by WGCNA

10.7717/peerj.8456/supp-4 Supplemental Information 4 Gene information of asthma development by WGCNA

10.7717/peerj.8456/supp-5 Supplemental Information 5 Source code for all figures

10.7717/peerj.8456/supp-6 Supplemental Information 6 Feature gene of asthma development by machine learning

10.7717/peerj.8456/supp-7 Supplemental Information 7 Correlation analysis of feature genes

## Additional Information and Declarations

### Data Availability

The following information was supplied regarding data availability: Raw data is available in the Supplementary Files.
